# Vicilin and legumin storage proteins are abundant in water and alkali soluble protein fractions of glandless cottonseed

**DOI:** 10.1038/s41598-021-88527-7

**Published:** 2021-04-28

**Authors:** Zhongqi He, Christopher P. Mattison, Dunhua Zhang, Casey C. Grimm

**Affiliations:** 1 USDA-ARS, Southern Regional Research Center, New Orleans, LA 70124 USA; 2 USDA-ARS, Aquatic Animal Health Research Unit, Auburn, AL 36832 USA

**Keywords:** Biotechnology, Computational biology and bioinformatics, Plant sciences

## Abstract

In this work, we sequentially extracted water (CSPw)- and alkali (CSPa)-soluble protein fractions from glandless cottonseed. SDS-Gel electrophoresis separated CSPw and CSPa to 8 and 14 dominant polypeptide bands (110–10 kDa), respectively. Liquid chromatography-electrospray ionization-tandem mass spectrometry identified peptide fragments from 336 proteins. While the majority of peptides were identified as belonging to vicilin and legumin storage proteins, peptides from other functional and uncharacterized proteins were also detected. Based on the types (unique peptide count) and relative abundance (normalized total ion current) of the polypeptides detected by mass spectrometry, we found lower levels (abundance) and types of legumin isoforms, but higher levels and more fragments of vicilin-like antimicrobial peptides in glandless samples, compared to glanded samples. Differences in peptide fragment patterns of 2S albumin and oleosin were also observed between glandless and glanded protein samples. These differences might be due to the higher extraction recovery of proteins from glandless cottonseed as proteins from glanded cottonseed tend to be associated with gossypol, reducing extraction efficiency. This work enriches the fundamental knowledge of glandless cottonseed protein composition. For practical considerations, this peptide information will be helpful to allow better understanding of the functional and physicochemical properties of glandless cottonseed protein, and improving the potential for food or feed applications.

## Introduction

With about 28 × 10^9^ kg or 124 million bales of cotton produced annually, cotton plant (*Gossypium hirsutum* L.) is an economically important crop known for fiber, cottonseed oil, and protein contents^1–3^. Currently, cotton fiber and cottonseed account for 85–90% and 10–15% of the value of the crop, respectively, while 150 kg of cottonseed is produced for every 100 kg fiber ginned. The cottonseed consists of approximately 16% oil, 45% meal, 25% hull, and 8% linters. The major components of defatted meal are carbohydrates and protein. Cottonseed protein comprises multiple polypeptides (i.e., various protein fractions) including seed storage proteins and proteins with other biological functions^4,5^. Cottonseed protein has great potential as a component of value-added industrial products and bio-active functional materials. The potential added-value products of cottonseed protein isolates include, but are not limited to, bio-based adhesives^6–10^, bioplastics and films^11,12^, antioxidant fractions/peptides^13,14^, and antibiotic peptides^15^.

The traditional variety of cottonseed contains the toxic terpenoid gossypol and is labeled “glanded cottonseed” as gossypol is deposited in scattered tissue structures called glands. Research efforts have been made to produce a new type of “glandless” cottonseed in which there is only trace gossypol content. Gossypol is a terpenoid produced and stored in the pigment glands of the typical cotton plant (about 1 weight percent (wt%) of dry matter of cottonseed)^16^. Thus, the traditional variety of cottonseed is also labeled as “glanded” cottonseed^17,18^. The presence of toxic gossypol limits cottonseed to mainly non-food applications^1^. Research has been directed towards eliminating or reducing gossypol from cottonseed (i. e., below 450 ppm free gossypol) to mitigate the toxic effects of gossypol. One notable genetically modified cultivar, TAM66274, has ultra-low gossypol cottonseed^16^. Another interesting genetically engineered cultivar is NuMex COT 15 GLS’ Glandless Cotton^17^. Cheng et al.^19,20^ demonstrated that the glandless cottonseed protein isolates could serve as wood adhesives. He et al.^21^ reported that glandless cottonseed protein-based adhesives had similar bonding performances as the glanded counterpart. In the latest study, He et al.^14^ comparatively examined the antioxidant activities of the water-soluble fractions of both glandless and glanded cottonseed protein. Cao et al.^18,22^ reported ethanol extracts from both glanded and glandless cottonseed kernels contained some bioactive ingredients.

Continued characterization of glandless cottonseed will enable improved understanding and food and feed utilization of glandless cottonseed protein. In this work, we isolated and separated glandless cottonseed proteins into water- and alkali-soluble fractions, analyzed their polypeptide profiles, and compared them to the peptide features of glanded cottonseed protein fractions.

## Results and discussion

### Polypeptide band features of glandless CSPw and CSPa on gradient SDS-PAGE

**Figure 1:**
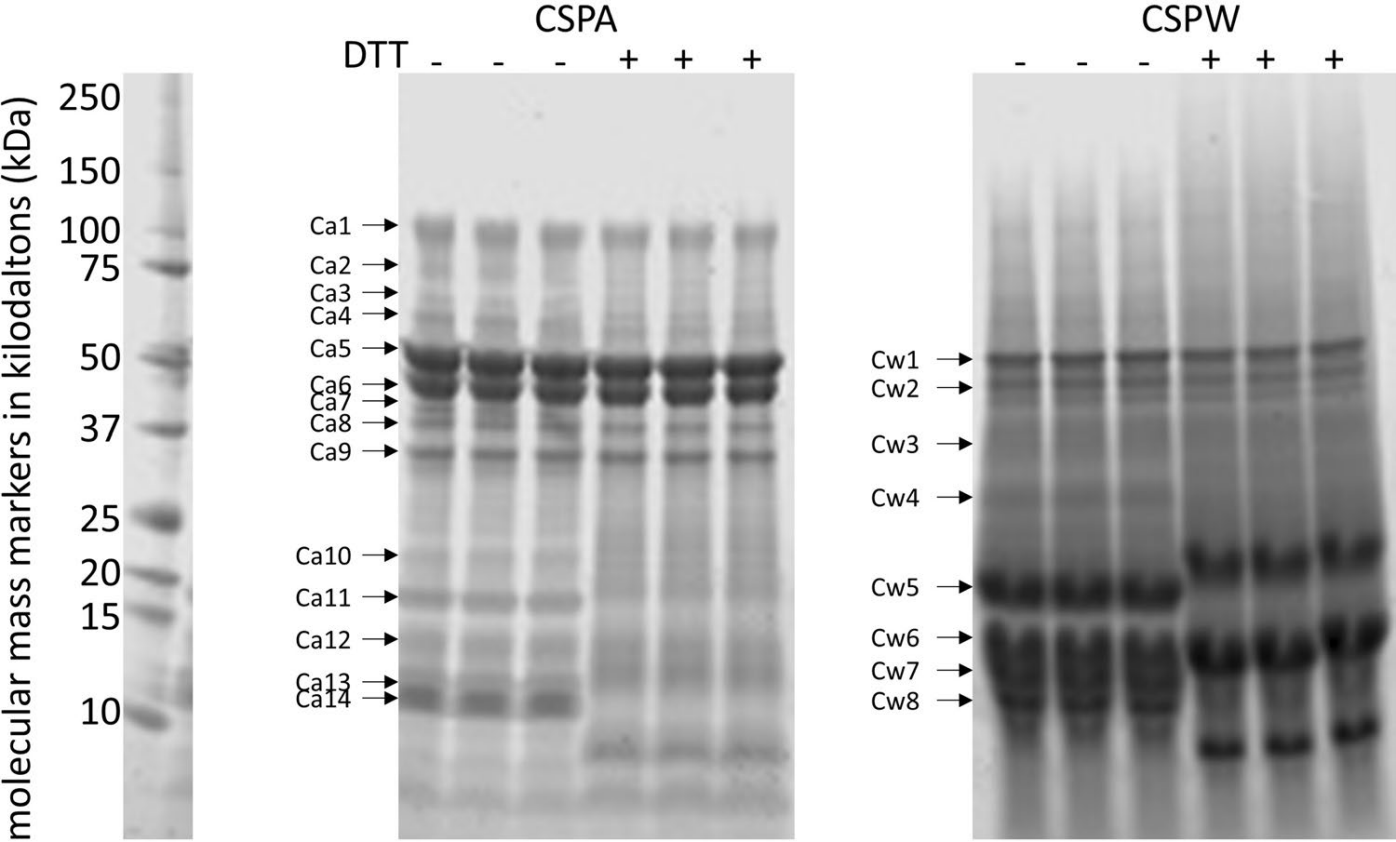
Gradient (10–20% Tricine) SDS-PAGE of water- (CSPw) and alkali- (CSPa) soluble cottonseed protein isolates with ( +) or without (−) dithiothreitol (DTT) treatment. Approximately 2 µg of protein were applied to each lane. Gels were visualized and protein band intensities were quantified using the 680 nm channel on a Li-Cor Odyssey infrared scanner and Image Studio software (LI-COR Biosciences, USA). The SDS-PAGE profile of corresponding glanded samples can be found in He et al.^4^.

**Figure 2:**
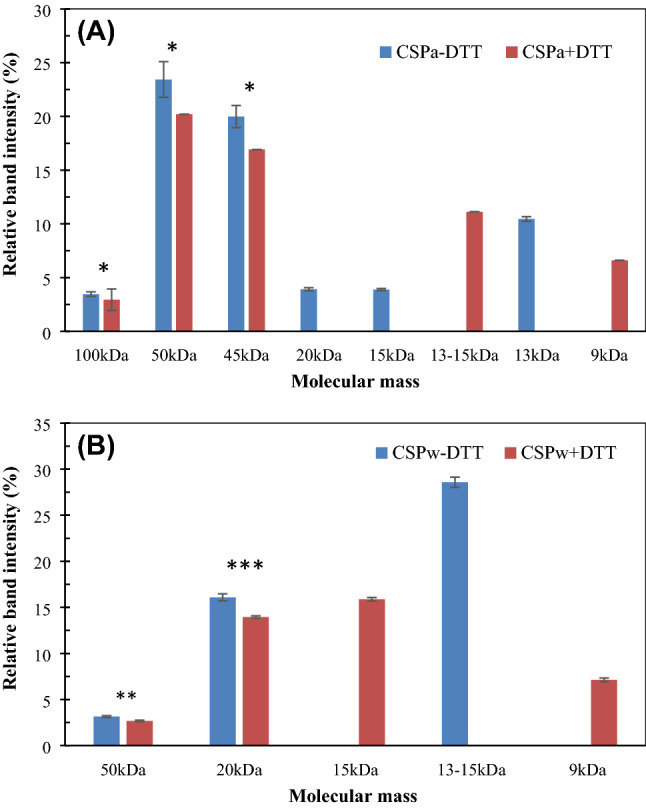
Relative protein band intensities as a percentage of total load with ( +) or without (−) dithiothreitol (DTT) treatment. **(A)** Water-soluble cottonseed protein (CSPw); **(B)** alkali-soluble cottonseed protein (CSPa). Data are presented as average ± standard deviation (n = 3). Symbol *, **, and *** indicate the band intensity with the same molecular weights between the two treatments significantly different at *p* = 0.05, 0.01, and 0.001, respectively.

SDS-gel electrophoresis separated CSPw and CSPa into 8 and 14 discernable polypeptide bands, respectively (Fig. 1, full-length gels and blots are included in a Supplementary Information file). The polypeptide bands ranged from over 100 to about 11 kDa with CSPa, but just over 50 to 10 kDa for CSPw. Among the 14 CSPa bands, Ca 5 and Ca 6 at 55 and 45 kDa were most abundant. Together, they accounted for 43% of the relative abundance of the total protein load (Fig. 2). In contrast, the three most abundant bands in the CSPw sample (Cw 5, 6 and 7) were about 20, 15 and 13 kDa. Together, they accounted for 45% of the relative abundance of the total CSPw protein load. These observations were generally consistent with previous data of glandless cottonseed proteins^14,23^.

Similar protein band patterns with minor differences were observed when comparing glandless to glanded protein extract. There were more CSPa bands, but less CSPw bands in the glandless sample when compared to a glanded sample with 7 CSPa and 12 CSPw bands^4^. The main difference in the glandless CSPa bands was the four molecular weight bands from 60 to 110 kDa (Ca 1 to 4) which were not observed in either the glanded CSPa fraction^4,24^ or in glanded whole cottonseed protein isolates^5,24,25^. The presence of gossypol could result in modified SDS-PAGE protein patterns due to the chemical reactivity of gossypol with proteins. Gossypol is known to react with the free ε-amino group of lysine^26^ , likely promoting the formation of protein aggregates that were more difficult to extract. In fact, protein yield was 2.5-fold greater from the glandless meal than that from the glanded meal^27^. The higher extraction efficiency not only increased the quantity of protein biomass, but likely increased the diversity of observed proteins.

The glandless CSPa and CSPw extracts were also resolved on SDS-PAGE with the reducing agent dithiothreitol (DTT) to identify proteins containing disulfide bonds (Fig. 1). The CSPa extract band pattern was clearly altered by the inclusion of DTT in the loading buffer. The migration of several lower molecular weight bands (e. g., Ca10, 11, 13, and 14) in the CSPa sample appeared to be altered by DTT. While none of the relatively higher molecular weight bands in CSPw around 50 kDa appeared to change following the addition of DTT, the migration of several lower molecular weight bands (e. g., Cw 4, 5, 7 and 8) in the CSPw fraction were altered. With the DTT treatment, the relative abundance of new bands appearing in the range of 9–15 kDa made up 18% and 23% of relative protein abundance, respectively, for CSPa and CSPw (Fig. 2). Ma et al.^25^ reported that, in the presence of the reducing agent β-mercaptoethanol, many additional minor bands appeared between 14 and 35 kDa, compared to SDS-PAGE of their glanded cottonseed protein samples in the absence of a reducing agent. The emergence of these minor bands under reducing condition suggests the presence of intermolecular disulfide bonds in cottonseed protein isolates. It is hard to distinguish the 58/59 kDa DTT sensitive bands in the gel in Fig. 1. We are not aware of any previous research on the relevant SDS-PAGE band patterns under reducing conditions for glandless cottonseed protein samples. Like our work, Delgado et al.^23^ presented the SDS-PAGE of water-, 0.5 M NaCl-, 70% ethanol-,and 0.1 NaOH-soluble fractions of glandless cottonseed protein without reducing agent treatment. More research is needed to find out if the less impact of reducing treatment on 58/59 kDa bands is due to much less disulfide-bonded proteins presented in the glandless samples, or some other causes.

### Peptide and protein profiles of CSPw and CSPa

The unique peptide count and their quantitative values are listed in spreadsheet SupTable1A and SupTable1B of the Supplementary Dataset File, respectively. In contrast to 2319 unique peptides and 70 proteins identified in the glanded sample^4^, there was a total of 5115 unique peptides that were matched to 336 proteins in the glandless extract. Therefore, the peptide diversity is wider for the glandless extracted than that for the glanded samples. The differences in observed peptide content provide support, but do not conclusively prove, the idea that extraction of proteins from glandless meal was more efficient than from the glanded meal^27^.

The molecular weight of proteins in the glandless cottonseed extracts ranged from 13 to 289 kDa, and several individual bands were compared to gene products or putative gene products of *Gossypium arboretum*, *G. hirsutum* or unspecified *Gossypium* species via UniPro (https://www.uniprot.org/uniprot/) and/or Conserved Domains in NCBI-BLASTp (https://blast.ncbi.nlm.nih.gov/Blast.cgi). Among them, 156 proteins appeared exclusively in CSPw bands, but only 19 proteins appeared exclusively in CSPa bands. There were 85 protein polypeptides that appeared only once in a unique CSPw band, and 22 proteins that appeared in all 8 CSPw bands. On the other hand, there were 78 protein polypeptides appearing uniquely in a single CSPa band, but 6 protein polypeptides that appeared in all 14 CSPw bands.

**Figure 3:**
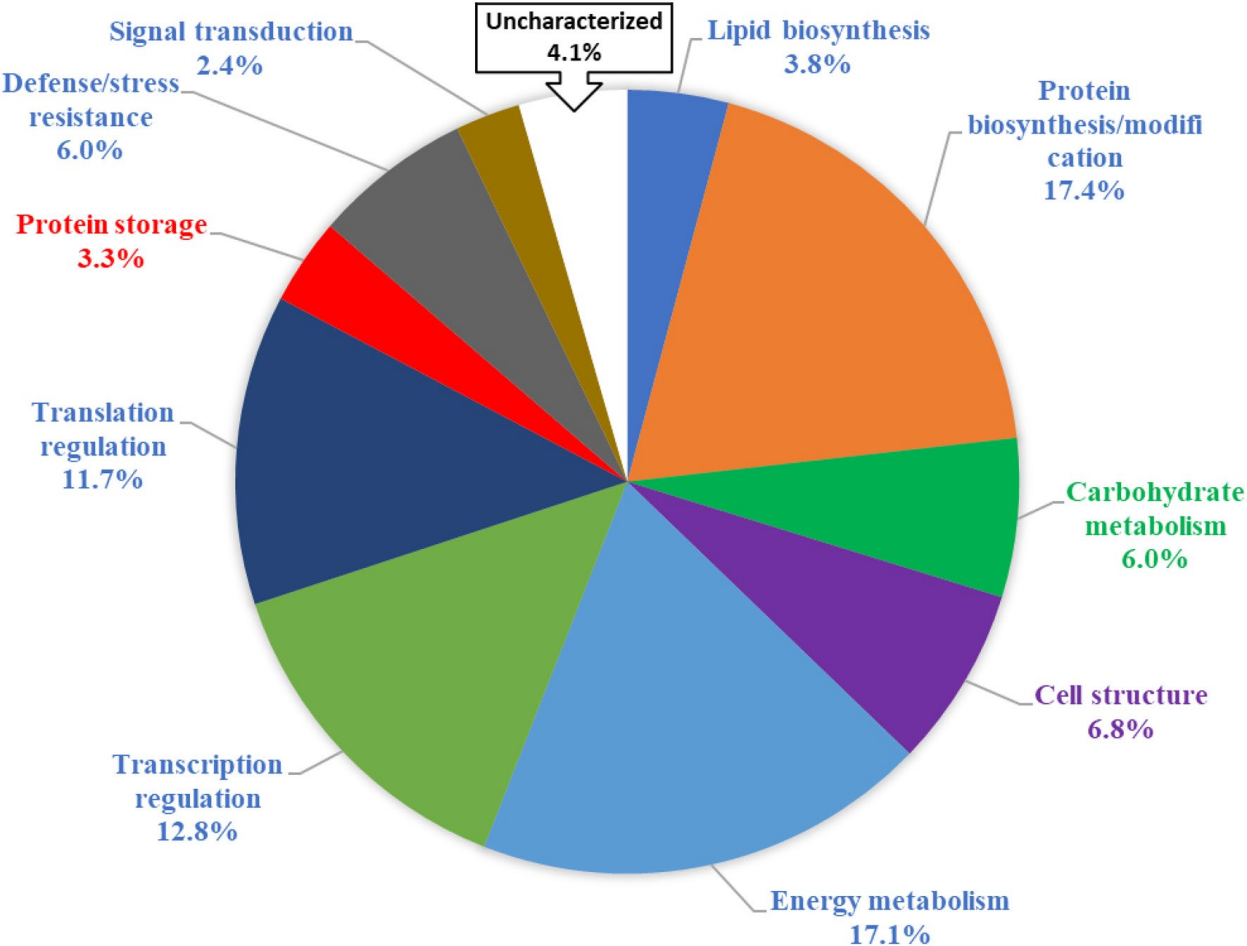
Biological functions and percentage of each class of proteins identified in glandless cottonseed predicted in accordance with Gene Ontology terms.

These proteins were functionally classified into 11 categories as protein storage, transporters, signal transduction, cell structure, transcription, translation, protein biosynthesis, protein metabolism, energy metabolism, antimicrobial activity, defense/stress, carbohydrate metabolism, fatty acid metabolism and protein species of unknown functions (Fig. 3). Biosynthesis/modification (17.4%), translation regulation (11.7%), transcription regulation (12.8%) and energy metabolism (17.1%) categories accounted for more than half of the proteins identified.

Peptides from the top 10 proteins accounted for about 25–53% of the protein biomass of a glandless CSPw band, and this was much lower than the percentages (84–100%) of any glanded CSPw band (Tables 1 and 2). This difference indicated a more diverse protein composition in glandless CSPw than in glanded CSPw. The percentages of the top 10 protein in glandless CSPa bands were generally high (> 90%), except that Ca12 (50.5%) and Ca14 (59%) were comparable to those in glanded CSPa bands. Vicilin proteins accounted for approximately 80% of glandless CSPa proteins including Ca12 (41.11%) and Ca14 (50.22%). Between the two, the abundance of vicilin-like antimicrobial peptides^28^ was higher in CSPa (4.52–12.93%) than in CSPw (2.65–6.36%). Legumins (2.39–12.45%) were the second most abundant proteins appeared in all 14 glandless CSPa bands, but not in top 10 of glandless CSPw. Indeed, legumin A and B accounted for only 0–0.88% and 0–1.9% of abundance, respectively, in glandless CSPw proteins which found in 7 and 6 of 8 CSPw bands, respectively. This observation was different from that of glanded CSPw fraction in which legumin A and B were the 1^st^ and 4^th^ most abundant, with a combined 22–53% of protein biomass (Table 1A).

**Table 1:** Top 10 proteins of CSPw per quantitative values (% of normalized total spectral strength of each band sample).

	MW, kDa	Cw1	Cw2	Cw3	Cw4	Cw5	Cw6	Cw7	Cw8				
**(A) Glandless cottonseed**													
Vicilin C72 (*G. hirsutum*)	70	14.11	12.48	5.88	7.40	19.82	12.96	24.67	14.46				
Vicilin GC72-A	71	10.17	14.27	5.79	5.72	4.07	23.91	15.42	10.58				
Vicilin C72 (*G. arboretum*)	70	12.24	11.44	5.41	0.00	0.00	0.00	0.00	0.00				
Vicilin-like antimicrobial peptides 2–1	62	2.90	2.84	3.70	1.78	2.54	3.54	2.64	1.76				
Phosphoglycerate kinase, cytosolic	42	1.56	1.61	1.33	1.68	3.94	1.52	1.76	2.47				
*Protein disulfide-isomerase #*	*56*	*1.04*	*1.1*3	*1.33*	*2.07*	*2.80*	*2.36*	*1.91*	2.65				
Vicilin-like antimicrobial peptides 2–2	55	1.14	2.08	2.66	2.27	1.14	0.51	1.17	0.88				
2S albumin storage protein	16	0.52	0.38	0.57	1.78	0.51	1.35	3.23	2.65				
*Embryonic DC-8 #*	*63*	*0.73*	*1.32*	*1.42*	*1.48*	*0.64*	*2.02*	*1.91*	*1.41*				
*Annexin D2-like protein #*	*36*	*1.66*	*1.23*	*4.08*	*0.79*	*1.52*	*0.00*	*0.15*	*1.06*				
Top 10 total		46.06	48.77	32.16	24.95	36.98	48.15	52.86	37.92				

	MW, kDa	Cw1	Cw2	Cw3	Cw4	Cw5	Cw6	Cw7	Cw8	Cw9	Cw10	Cw11	Cw12
**(B) Glanded cottonseed **^4^													
Legumin A	58	13.39	20.29	21.32	24.80	26.55	33.93	23.62	22.88	23.26	23.14	32.20	31.08
Vicilin C72 (*G. hirsutum*)	70	30.71	23.91	27.94	23.20	17.70	20.54	25.20	26.27	22.48	24.79	23.73	21.62
Vicilin GC72-A	71	18.90	20.29	17.65	20.00	22.12	19.64	21.26	20.34	20.16	20.66	18.64	24.32
Legumin B	59	8.66	10.87	16.18	19.20	18.58	18.75	18.11	18.64	17.83	22.31	16.95	21.62
Vicilin-like antimicrobial peptides 2–1	62	6.30	5.80	11.03	4.80	7.96	1.79	4.72	5.08	2.33	3.31	5.08	0.00
Vicilin C72 (*G. arboretum*)	70	5.51	2.90	2.94	0.80	1.77	2.68	1.57	0.00	1.55	0.83	0.85	0.00
2S albumin storage protein	16	0.00	0.00	0.74	0.80	0.00	0.00	0.00	2.54	0.00	0.00	0.00	1.35
**Transcription initiation factor TFIID subunit 1-B-like protein #**	**111**	**0.00**	**2.17**	**0.00**	**0.00**	**0.88**	**0.00**	**0.00**	**0.00**	**0.78**	**0.83**	**0.00**	**0.00**
Protein lin-54	82	0.79	0.72	1.47	0.00	0.00	0.00	0.00	0.00	0.78	0.00	0.00	0.00
Vicilin-like antimicrobial peptides 2–2	55	0.00	0.00	0.00	0.80	0.00	0.89	0.79	0.00	0.78	0.83	0.00	0.00
Top 10 total		84.25	86.96	99.26	94.40	95.58	98.21	95.28	95.76	89.92	96.69	97.46	100.0

Italics: # Not found in glanded protein; Bold: # not found in glandless protein.

**Table 2:** Top 10 proteins of CSPa per quantitative values (% of normalized total spectral strength of each band sample).

	MW, kDa	Ca1	Ca2	Ca3	Ca4	Ca5	Ca6	Ca7	Ca8	Ca9	Ca10	Ca11	Ca12	Ca13	Ca14
**(A) Glandless cottonseed**															
Vicilin GC72-A	71	32.78	33.77	31.51	30.76	19.19	36.94	32.16	23.68	31.92	27.31	22.37	15.98	36.26	18.83
Vicilin C72 (*G. hirsutum*)	70	26.01	21.71	21.89	24.49	38.38	22.72	23.28	25.64	24.53	28.88	31.18	17.77	20.88	27.95
Vicilin C72	70	23.26	20.22	20.38	21.55	31.98	22.72	22.45	25.64	24.21	22.59	29.46	0.00	19.12	0.00
Legumin A	58	6.59	8.53	8.49	7.18	2.65	6.34	7.37	5.09	5.35	3.73	3.44	2.33	7.91	1.64
Vicilin-like antimicrobial peptides 2–1	62	3.85	4.82	5.09	4.05	2.96	4.79	3.35	7.63	3.62	2.16	2.58	5.03	3.96	2.54
Legumin B	59	2.38	2.78	3.96	4.24	0.78	1.70	3.35	2.35	4.09	3.73	3.23	3.05	1.32	0.75
2S albumin storage protein	16	0.18	1.11	0.94	2.76	0.78	0.31	1.68	0.59	0.47	3.73	0.65	2.51	4.40	5.68
Vicilin-like antimicrobial peptides 2–2	55	0.37	1.11	0.94	0.55	0.00	0.00	0.50	0.78	0.16	0.59	0.22	2.33	0.22	0.90
*Asparticase#*	*60*	*0.37*	*0.00*	*0.00*	*0.37*	*0.47*	*1.08*	*0.34*	*0.20*	*0.31*	*0.00*	*0.43*	*1.26*	*0.00*	*0.30*
*60S ribosomal L10a-1-like protein#*	*25*	*0.55*	*0.93*	*0.57*	*0.55*	*0.00*	*0.00*	*0.67*	*0.59*	*0.31*	*0.00*	*0.43*	*0.18*	*0.00*	*0.00*
Top 10 total		96.34	94.99	93.77	96.50	97.19	96.60	95.14	92.17	94.97	92.73	93.98	50.45	94.07	58.59

	MW, kDa	Ca1	Ca2	Ca3	Ca4	Ca5	Ca6	Ca7							
**(B) Glanded cottonseed**^4^															
Vicilin C72 (*G. hirsutum*)	70	35.56	18.12	14.58	14.76	26.78	20.32	25.28							
Vicilin C72 (*G. arboretum*)	70	39.87	18.49	13.54	14.51	23.45	20.55	22.68							
Vicilin GC72-A	71	15.09	45.78	15.48	10.91	11.35	25.64	10.60							
Legumin A	58	2.80	8.56	38.39	26.70	11.80	15.94	27.51							
Legumin B	59	2.48	2.73	6.40	29.40	18.61	8.55	5.95							
Vicilin-like antimicrobial peptides 2–1	62	1.08	1.86	3.42	0.26	1.06	1.39	2.04							
Late embryogenesis abundant protein D-19	11	0.22	0.00	0.89	0.64	0.61	0.69	0.93							
**ATP synthase subunit beta #**	**60**	**0.43**	**0.25**	**0.74**	**0.13**	**0.76**	**0.46**	**0.37**							
Heat shock protein 70	71	0.00	0.12	1.04	0.00	1.06	0.00	0.37							
Elongation factor 1-alpha	49	0.65	0.25	0.60	0.13	0.00	0.23	0.56							
Top 10 total		98.17	96.16	95.09	97.44	95.46	93.77	96.28							

Italics: # Not found in glanded protein; Bold: # not found in glandless protein.

The cotton 2S albumin was also observed in both CSPw and CSPa fractions of glandless cottonseed protein, but was not in the top 10 list of the glanded CSPa fraction. In contrast, another storage protein, the late embryogenesis abundant protein D-19, appeared in glanded CSPa sample. The lipid bodies-related storage protein oleosin with molecular weights of 16.4 and 18.2 kDa was also found in both CSPw and CSPa fractions, but with lower abundances than other storage proteins and was not in the top 10 for either cottonseed sample.

Peptides from three proteins of known function (cytosolic phosphoglycerate kinase, embryonic DC-8, annexin D2-like protein) and two proteins of known function (asparticase and 60S ribosomal L10a-1-like protein) were found in the top 10 of glandless CSPw and CSPa, respectively. Interestingly, none of them were found in the 90 proteins identified in glanded cottonseed samples. Similarly, two proteins of known function appeared in CSPw (transcription initiation factor TFIID subunit 1-B-like protein, and protein lin-54) and were not found in the total list of 336 proteins of glandless cottonseed. However, two (heat shock protein 70, and elongation factor 1-alpha) of the three functional proteins in the top 10 of glanded CSPa, were observed, but were not in the top 10, in the 336 proteins of glandless cottonseed.

### Comparison of peptide fragments of legumins in glandless and glanded proteins

**Figure 4:**
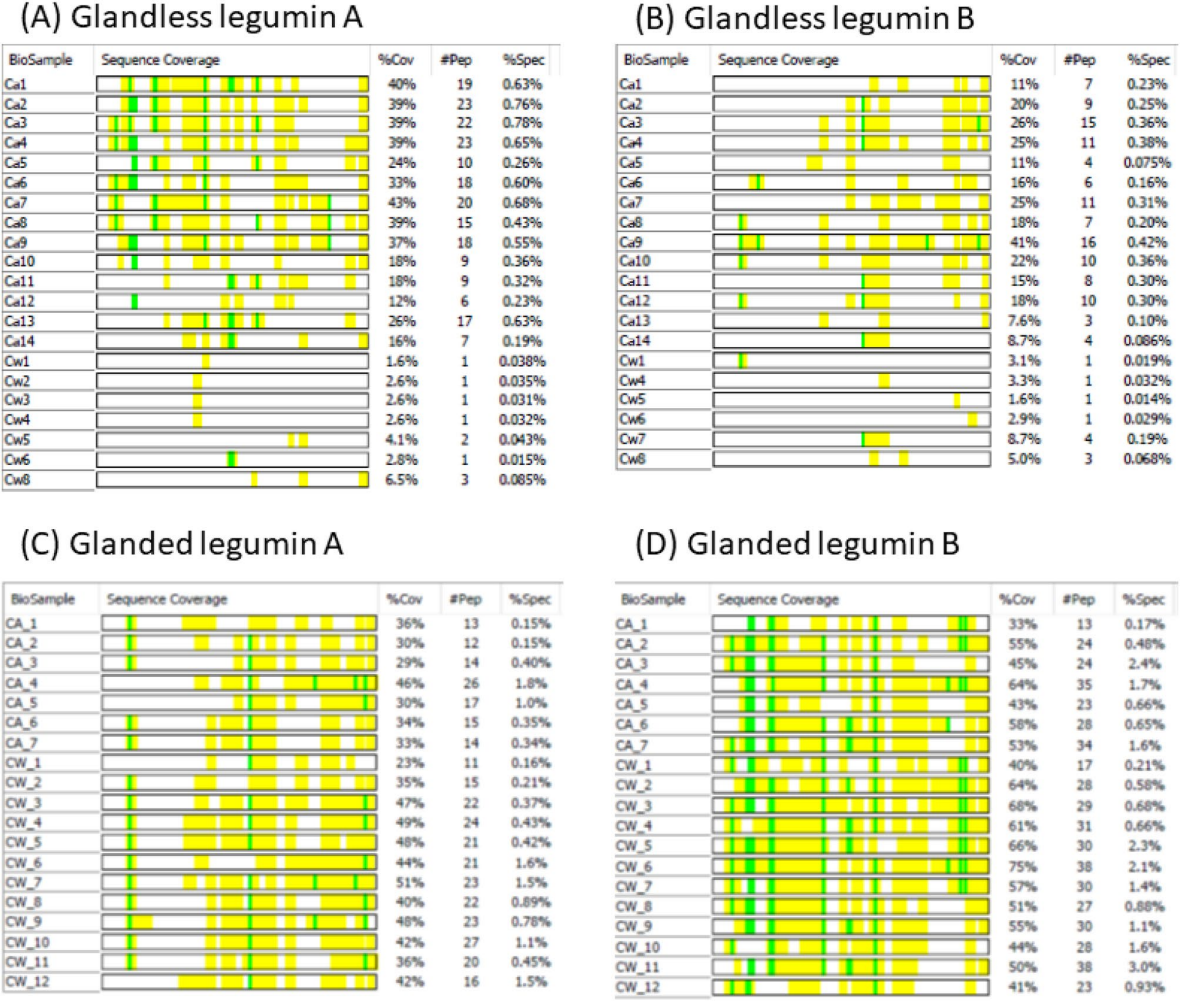
Sequence coverage of peptide fragments of legumins in glandless and glanded samples. The identified peptides are highlighted in yellow and the post-translational modification in green. The columns “% Cov1”, “# Pep” and “% Spec” show the % of the identified fragment strings in the full length of the protein sequence, number of peptide fragments, and their relative abundance in each band, respectively. Data of glanded sample are from the work described in He et al.^4^.

Peptide fragments of legumin storage proteins were identified in multiple bands in both CSPw and CSPa from glandless and glanded cottonseed, and were further analyzed to compare the number of observed peptides in each band (Fig. 4). The molecular weight of legumin A is approximately 58 kDa. While legumin A peptides were identified in all glandless CSPa bands from 100 to 9 kDa, the percentage of sequence coverage and number of peptide fragments were generally higher in high molecular weight gel bands than in low molecular CSPa bands. This observation indicated that legumin A of glandless cottonseed was mainly present in CSPa bands as whole protein, multiple and/or long peptide fragments. In contrast, there were just 1, 2 or 3 peptide fragments appearing in 7 of the 8 CSPw bands, suggesting degraded legumin A peptides were present in the CSPw fraction of glandless cottonseed protein. The distribution pattern of legumin B (approximately 59 kDa) was similar to that of legumin A. However, the number of peptide fragments and percent of sequence coverage of legumin B were lower than legumin A in glandless cottonseed. In contrast, peptides of both legumin A and B from glanded cottonseed were distributed in both CSPw and CSPa bands with similar patterns. The high sequence coverage suggested the two legumins were present as whole proteins or nearly full-length polypeptides in the CSPa and CSPw fractions of glanded cottonseed proteins.

Multiple bands from a single protein could be due to three mechanisms: (1) degradation during protein isolation and post treatment, (2) contamination from gels, and (3) indigenous isoforms (including post-translational protein modification)^4^. While the first and second mechanisms are possible, the systematic difference in the sequence coverage patterns observed between glandless and glanded cottonseed protein suggested the presence of isoforms. In the investigation of genomically biased accumulation of seed storage proteins in allopolyploid cotton, Hu et al.^29^ proposed that legumin isoforms may be formed through a series of modifications, including proteolytic cleavage and peptide degradation. Specifically, they found 83 spots as legumin A, and 27 spots as legumin B out of 155 identified in a 2-D SDS-PAGE image of cottonseed protein. Isoform analysis through peptide mapping indicated that the 30-kDa polypeptide of legumin A derived from the C-terminal fragment of the 58 kDa pre-propolypeptide. Legumin isoforms are commonly distributed at molecular mass of 30 kDa, 17–20 kDa, and 11–12 kDa as legumin A and at molecular weights of 11–13 kDa as legumin B. Therefore, the differences in legumins between glandless and glanded cottonseed proteins may indicate that lower levels (abundance) and/or types of legumin isoforms were present in glandless cottonseed. It remains to be determined if legumin protein expression could be impacted or not by the genetic engineering of the glandless Upland cotton (*G. hirsutum* L.) cultivar, which carried the incomplete dominant glandless *Gl2*^*e*^ allele introduced from *G. barbadense*^17^.

### Comparison of vicilin peptide fragments in glandless and glanded proteins

**Figure 5:**
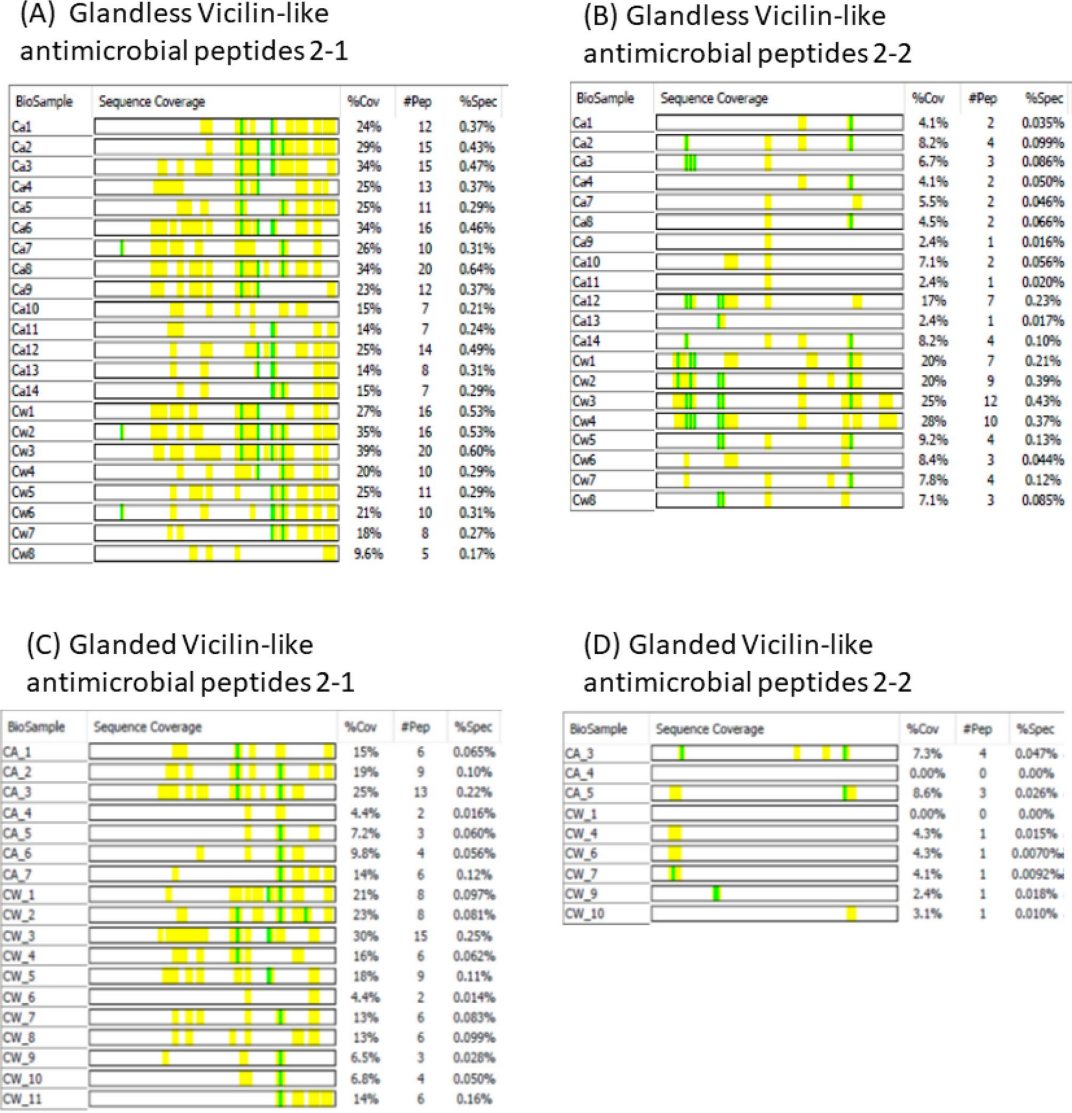
Sequence coverage of peptide fragments of vicilin-like antimicrobial peptides in glandless and glanded samples. The identified peptides are highlighted in yellow and the post-translational modification in green. The columns “% Cov1”, “# Pep” and “% Spec” show the % of the identified fragment strings in the full length of the protein sequence, number of peptide fragments, and their relative abundance in each band, respectively. Data of glanded sample are from the work described in He et al.^4^.

Like legumins, vicilins are primary storage proteins of cottonseed with multiple types and isoforms^29,30^. Similar to glanded samples, five types of vicilin proteins were identified in glandless cottonseed protein with a wide range of molecular weights (100–9 kDa). Two vicilin-like antimicrobial peptides possess potential antifungal activity in plant development in cropping management and function against important yeasts in medical mycology for medical application^31,32^. The distribution of these peptides in glandless and glanded cottonseed proteins was compared (Fig. 5). No apparent difference in peptide sequence or protein coverage pattern was observed between glandless and glanded vicilin proteins. However, in contrast to legumins, both vicilin-like antimicrobial peptides 2–1 and 2–2 seemed more abundant in glandless samples than in the corresponding glanded CSPa and CSPw samples. As the glandless cotton cultivar is depleted in gossypol in cottonseed, the increasing level of vicilin-like antimicrobial peptides may be a compensation mechanism to offset the beneficial role of gossypol’s antimicrobial properties during germination and young plantlet development of the glandless cotton^32,33^.

Compared to the distribution of antimicrobial peptide 2–2 fragments, more antimicrobial 2–1 peptides were identified in SDS-PAGE bands in both glandless and glanded cottonseed proteins. Interestingly, the peptide 2–1 fragments were concentrated in the C-terminus, but more SDS-PAGE bands were observed to contain N-terminal fragments in vicilin-like antimicrobial peptide 2–2. Chung et al.^34^ separated cottonseed protein into four major cotton seed basic protein fractions by a series of chromatographic procedures, and tested their in vitro antifungal activity. They suggested that those small molecular weight cysteine-rich vicilin-like peptides (approximately 16–20 kDa) with in vitro antifungal activity were from an approximately 70 kDa pre-proprotein. Furthermore, those vicilin-like antibacterial proteins (9–11 kDa) corresponded to N-terminally truncated forms of cysteine-rich small vicilin proteins (e.g., the 16.3 kDa protein). Their work seems consistent with our observation of the presence of both C-terminal and N-terminal enriched peptide 2–1 and 2–2. Hu et al.^29^ found that small isoforms of vicilin A (49, 35, 11, and 17 kDa) and vicilin B (49 and 17 kDa) were derived from the 70 kDa vicilin A and vicilin B pre-propolypeptides through the cleavage of signal peptides together with the N-terminal fragments, respectively, and further proposed that protein modifications (e.g., glycosylation, phosphorylation, acetylation, and methylation) likely contributed to the formation of these vicilin isoforms. Thus, there is rational and physiological justification for increased antimicrobial vicilin-like peptide production through post-translational cleavage of vicilin preprotein from glandless cotton (lacking in gossypol). The increased relative amount of antimicrobial proteins regarding glandless cottonseed protein samples would enhance its value in post-harvest products such as nutrient foods, food additives, and animal feed with better shelf life^14,23,35,36^ although the antimicrobial effect in the processed industrial products needs to be further confirmed.

### Comparison of peptide fragments of 2S albumin and oleosin in glandless and glanded protein

**Figure 6:**
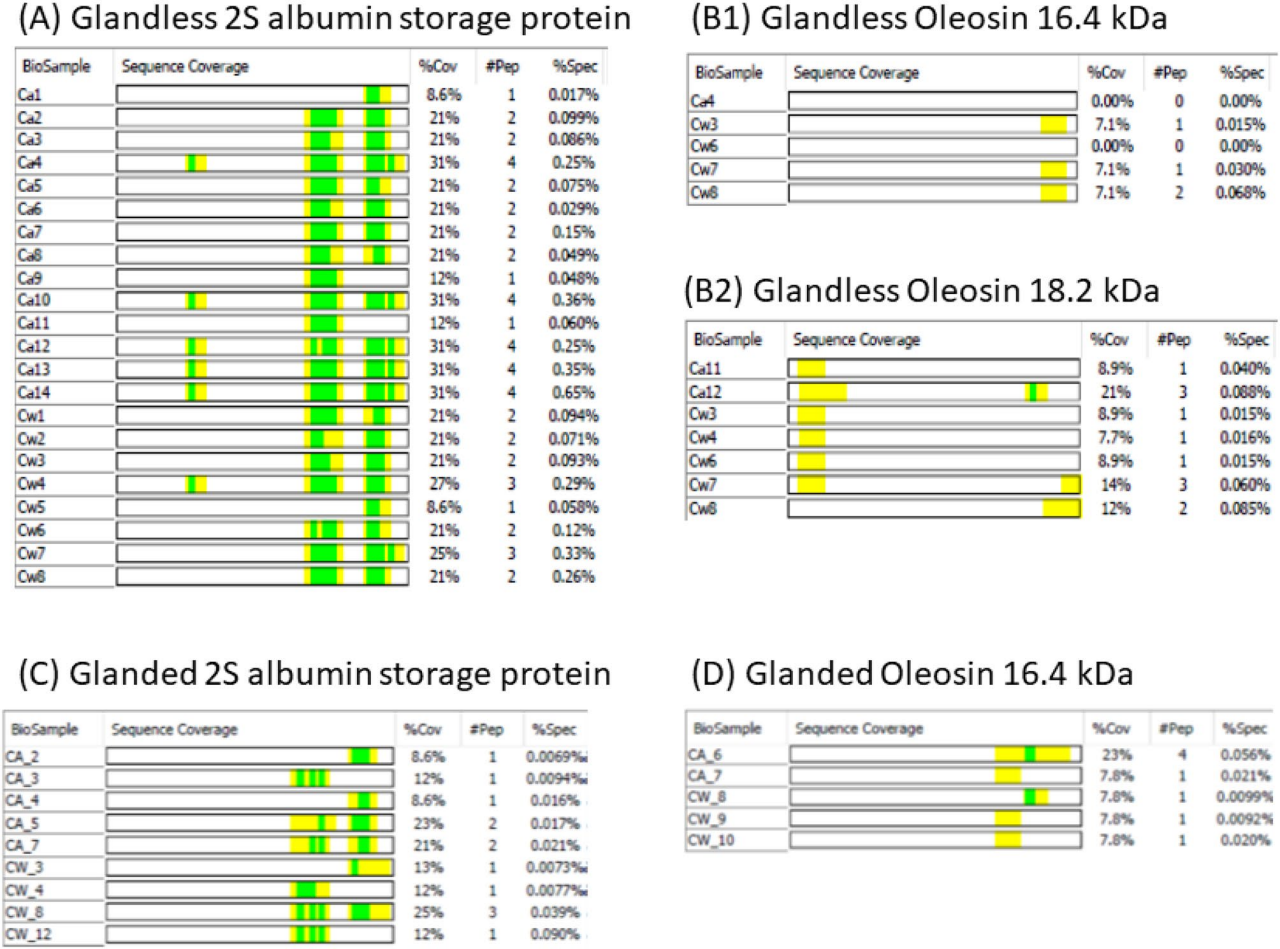
Sequence coverage of peptide fragments of 2S albumin and oleosin in glandless and glanded samples. The identified peptides are highlighted in yellow and the post-translational modification in green. The columns “% Cov1”, “# Pep” and “% Spec” show the % of the identified fragment strings in the full length of the protein sequence, number of peptide fragments, and their relative abundance in each band, respectively. Data of glanded sample are from the work described in He^4^.

Both 2S albumin and oleosin are storage proteins. Peptides matching with 2S albumin were found in all 22 CSPa and CSPw bands of glandless cottonseed protein, but only 9 of 19 bands of glanded samples (Fig. 6). Generally, the peptide strings (1–4 fragments) from glandless cottonseed protein were longer than the peptide sequence coverage of the glanded peptide fragments. In addition, the 2S albumin peptides from glanded cottonseed samples were only observed in the C-terminus of the protein. Sharing the homology to potent antifungal proteins from cheeseweed seeds, these small 2S albumin peptide fragments could potentially be used by cotton to form multiple antimicrobial molecules against a wide variety of pathogens^37^ during cottonseed germination.. By the way, more oxidation and carbamidomethyl (CAM) modification were observed with the 2S albumin fragments in glandless bands (up to 9 modifications in glandless sample vs 6 or less modifications in glanded sample shown in green in Fig. 6. However, CAM was a deliberate post-translational modification introduced to cysteine residues by reacting with iodoacetamide during sample preparation so that it was not indicative of a plant directed effort. The oxidation modifications could also part of sample preparation and not likely to be plant directed.

There were two types of lipid body-related oleosins with molecular weights of 16.4 kDa and 18.2 kDa in glandless samples, but only one type in glanded cottonseed protein (Fig. 6). They were relatively less abundant (not in the top 10), and only found in a few SDS-PAGE bands. The fragments from the 16.4 kDa oleosin were from the C-terminus of the protein in both glandless and glanded cottonseed samples even though the observed peptides were different. In contrast, 5 of 6 bands from the N-terminus of the 18.2 kDa oleosin were observed. Tzen et al.^38^ reported that the difference between maize 16 kDa and 18 kDa olesin proteins can be explained by an extra amino acid sequence of 2 kDa at the C-terminus of the 18 kDa. Santiago et al.^39^ found that coconut oleosin was composed of various isoforms with molecular weights of 48.78, 35.12, 32.39, 28.15, 22.06, and 16.13 kDa from SDS-PAGE analysis. Similarly, the possible presence of multiple isoforms of cottonseed oleosin can be seen in Fig. 5 B and D. The olesin protein in glandless cottonseed was dominated by the 18.4 kDa form, while the majority, if not all, of glanded oleosin was the 16.2 kDa form. Oleosins mainly act as oil body structural proteins and are important for the biogenesis, stability, and dynamics of the organelle^40^. Further research on the two oleosins in the two types of cottonseeds may shed new light into their roles in metabolism and cotton development.

## Conclusions

This work reported the polypeptide profile of water and alkali soluble protein fractions of glandless cottonseed. SDS-Gel electrophoresis separated CSPw and CSPa to 8 and 14 dominant protein bands, respectively. LC–ESI–MS/MS of the 22 protein bands identified a total of 5115 unique peptides that were matched to 336 proteins. While the majority of peptides were matched to vicilin and legumin storage proteins, peptides from other functional and uncharacterized proteins were also detected. Compared to the polypeptide profile of the glanded cottonseed protein in literature, glandless cottonseed protein possessed relatively lower levels (abundance) and types (diversity) of legumin isoforms, but higher levels and more fragments of vicilin-like antimicrobial peptides. This work enriched the fundamental knowledge of glandless cottonseed protein composition, thus being helpful in better understanding the functional and physicochemical properties of glandless cottonseed protein for food and feed applications.

## Materials and methods

### Cottonseed protein products

Defatted cottonseed meal from glandless variety “NuMex 15 GLS” was provided by Cotton, Inc. (Cary, NC, USA)^41^. A two-step extraction and precipitation procedure was applied to obtained the water soluble (CSPw) and alkali soluble (CSPa) glandless cottonseed protein fractions from the defatted meal^24,42^. Briefly, the water- (CSPw) and alkali-(CSPa) soluble protein fractions in the defatted cottonseed meal were sequentially extracted by water (naturally around pH 7.0) and 0.015 M NaOH, and then precipitated at pH 4.0 and 7.0, respectively, by pH adjustment with 1 M HCl.. Both fractions were freeze-dried and kept at -20 °C until use. It should be aware that the sequential extraction of the cottonseed protein was a relative measure of different fractions in defatted meal. This is because, prior to defatting, cottonseeds, like other oilseeds, are generally subjected to a “cooking” process which might make some protein denatured and aggregated, affecting their relative solubility^43,44^. The protein contents of CSPw and CSPa were about 79% and 97%, respectively, converted from the total N content using d using a conversion factor of 5.5^45^. To detect the presence of protein disulfide bonds dithiothreitol (DTT, Sigma-Aldrich, St. Louis, MO, USA) at 4 mM final concentration was added to CSPw and CSPa samples (2 µg) in 50 mM ammonium bicarbonate buffer (pH 8.0) at 37 °C for 15 min. Prior to trypsin digestion and LC–MS/MS analysis, reduced protein samples were alkylated in the dark with 15 mM iodoacetamide in 50 mM ammonium bicarbonate buffer (pH 8.0) for 30 min at room temperature. Iodoacetamide was then quenched by the addition of 4 mM DTT.

### Sodium dodecyl sulphate–polyacrylamide gel electrophoresis (SDS-PAGE)

CSPw and CSPa samples (2 µg/lane) were solubilized in 4X NuPAGE LDS Sample Buffer (pH 8.5) (ThermoFisher Scientific, USA), with or without DTT as above, and heated at 55 °C for 5 min. Following a brief centrifugation step to remove particulates, samples were electrophoresed for 90 min at constant voltage (125 V) on Novex 10–20% Tricine Mini Protein Gels (ThermoFisher Scientific, USA) using an XCell SureLock Mini-Cell (ThermoFisher Scientific, USA) apparatus with prestained Precision Plus molecular weight standards (Bio-Rad, USA)^46^. Following electrophoresis, gels were rinsed and stained with SimplyBlue SafeStain (ThermoFisher Scientific, USA) according to manufacturer’s instructions. Gels were visualized and protein band intensities were quantified using the 680 nm channel on a Li-Cor Odyssey infrared scanner and Image Studio software (LI-COR Biosciences, USA). The relative band intensity of a band was calculated by the percentage of that specific band intensity out of the sum of all band intensity values, and used to represent the relative protein content of that band in the total protein mass).

### Liquid chromatography–electrospray ionization–tandem spectrometry (LC–ESI–MS/MS)

Another set of CSPw and CSPa samples (10 µg/lane for six lanes each sample) were subjected to SDS-PAGE under the same conditions as described above. Individual bands excised from the multiple SDS-PAGE lanes were pooled and subjected to in-gel trypsin digestion^42^. The fragments in the digestion samples were analyzed by LC–ESI–MS/MS by UAB Mass Spectrometry/Proteomics Shared Facility (University of Alabama at Birmingham, Birmingham, Alabama, USA). The data were acquired with Bruker UltraFlex III MALDI ToF/ToF. The tandem mass spectral data generated were processed with SEQUEST and searched by Mascot against protein databases. The quantitative values of the normalized total ion current (TIC) of the peptide MS fragments were used as a relative measurement of the observed peptide in the gel bands. Scaffold (version Scaffold_4.11.0, Proteome Software Inc., Portland, OR) was used to validate MS/MS based peptide and protein identifications. The molecular function of individual proteins was searched via UniPro (https://www.uniprot.org/uniprot/) and/or Conserved Domains in NCBI-BLASTp (https://blast.ncbi.nlm.nih.gov/Blast.cgi). In addition to the specific functions, per the online search results, these proteins were grouped into 11 categories (Gene Ontology terms) for general identification.

### Experimental design and statistical analysis

Relative abundance of the major protein fractions separated by SDS-PAGE were calculated as their band intensities relative to the total band intensity of each lane. The lanes were measured and data were presented as the average with standard deviation (n = 3).

Same gel bands of six SDS-PAGE lanes were pooled as one sample for LC–ESI–MS/MS. For MS data treatments, peptide identifications were accepted if they could be established at greater than 80.0% probability by the Peptide Prophet algorithm with Scaffold delta-mass correction. Protein identifications were accepted if they could be established at greater than 99.0% probability and contained at least 2 identified peptides. Protein probabilities were assigned by the Protein Prophet algorithm^47^. Proteins that contained similar peptides and could not be differentiated based on MS/MS analysis alone were grouped to satisfy the principles of parsimony.

## Supplementary Information

Supplementary Table.

Supplementary Figure.
