# Supplementary material for: Vicilin and legumin storage proteins are abundant in water and alkali soluble protein fractions of glandless cottonseed
Source: Sci Rep. 2021 Apr 28;11:9209. doi: 10.1038/s41598-021-88527-7 (PMC8080652; doi:10.1038/s41598-021-88527-7)

Vicilin and Legumin Storage Proteins are Abundant in Water and Alkali Soluble Protein Fractions of Glandless Cottonseed

Zhongqi He [1,*], Christopher P. Mattison[1], Dunhua Zhang [2], and Casey Grimm [1]

[1] USDA-ARS, Southern Regional Research Center, New Orleans, LA 70124, USA.

[2] USDA-ARS, Aquatic Animal Health Research Unit, Auburn, AL 36832, USA

* Corresponding author. Email address: zhongqi.he@ars.usda.gov; zhongqi.he@usda.gov (ZH)

Image of the full-length SDS-PAGE gels and blots

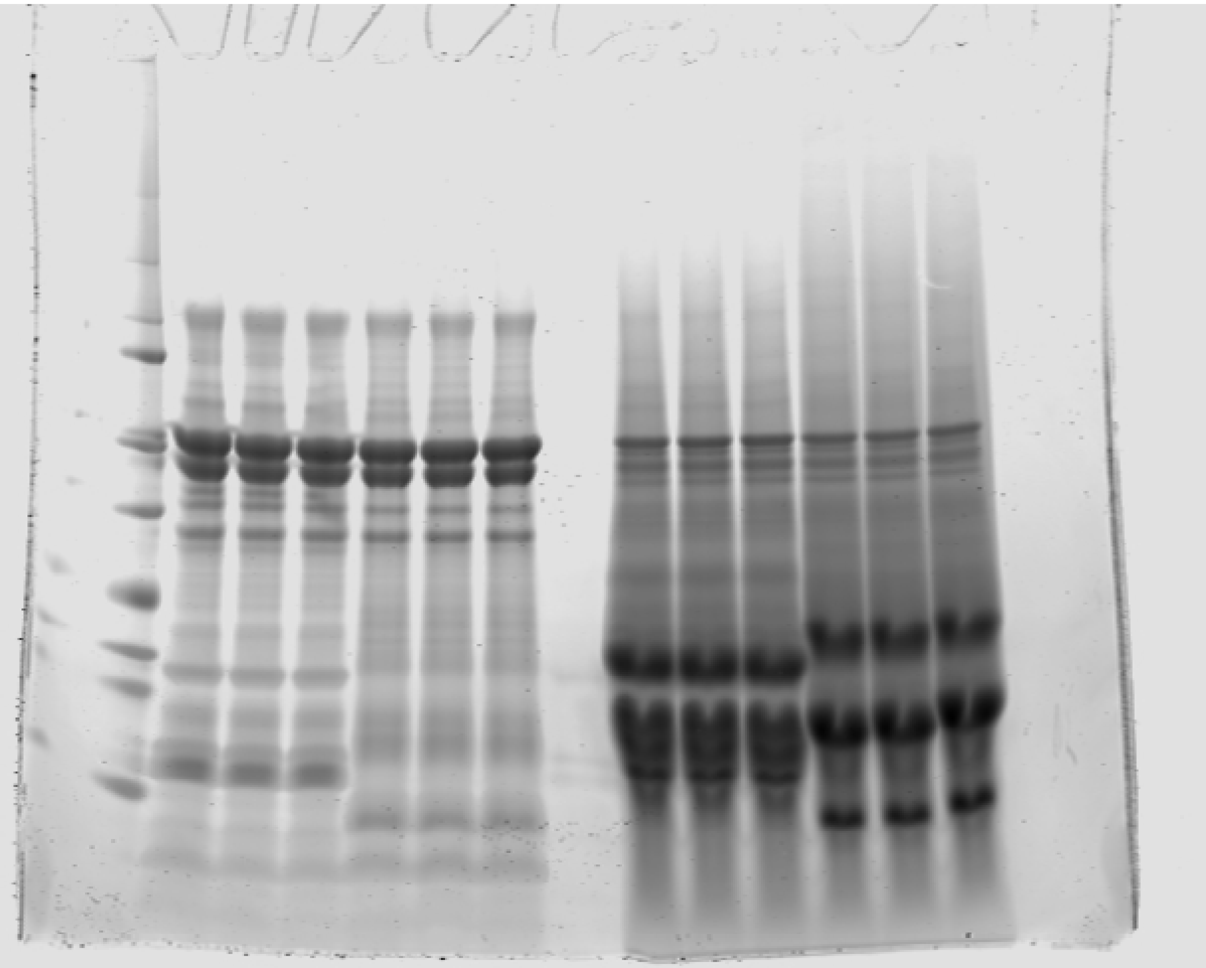

Supplement: Supplementary file 2 — Supplementary Figure. [file 41598_2021_88527_MOESM2_ESM.pdf]
